# *Pasteurella multocida* capsular: lipopolysaccharide types D:L6 and A:L3 remain to be the main epidemic genotypes of pigs in China

**DOI:** 10.1186/s44149-021-00031-7

**Published:** 2021-11-02

**Authors:** Songtao Liu, Lin Lin, Hao Yang, Wenqing Wu, Long Guo, Yue Zhang, Fei Wang, Xueying Wang, Wenbo Song, Lin Hua, Wan Liang, Xibiao Tang, Huanchun Chen, Zhong Peng, Bin Wu

**Affiliations:** 1 State Key Laboratory of Agricultural Microbiology, College of Veterinary Medicine, Huazhong Agricultural University, Wuhan, China; 2 MOST International Research Center for Animal Disease, Cooperative Innovation Center for Sustainable Pig Production, Huazhong Agricultural University, Wuhan, China; 3 MARA Key Laboratory of Prevention and Control Agents for Animal Bacteriosis, Animal Husbandry and Veterinary Institute, Hubei Academy of Agricultural Sciences, Wuhan, China

**Keywords:** *Pasteurella multocida*, Capsular and LPS genotypes, Virulence factor-encoding genes, Antimicrobial susceptibility, Pigs, China

## Abstract

*Pasteurella multocida* is a leading cause of respiratory disorders in pigs. This study was designed to understand the genotypical and antimicrobial resistant characteristics of *P. multocida* from pigs in China. To achieve this, we briefly investigated 158 *P. multocida* isolates from pigs with respiratory disorders in China between 2019 and 2020. Genotyping through multiplex PCR assays assigned these 158 isolates into capsular genotypes A (60.13%, 95/158), D (35.44%, 56/158), F (4.43%, 7/158), and/or lipopolysaccharide (LPS) genotypes L3 (28.48%, 45/158) and L6 (66.46%, 105/158). In addition, eight isolates (5.06%, 8/158) were found to be nontypable using the LPS genotyping method. When combining the capsular genotypes and the LPS genotypes, D: L6 (34.81%, 55/158) and A: L6 (31.65%, 50/158) were the predominant genotypes, followed by A: L3 (24.05%, 38/158). PCR detection of virulence factor-encoding genes showed that over 80% of the isolates were positive for *exbB*, *tonB*, *exbD*, *ompH*, *ptfA*, *fimA*, *sodA*, *sodC*, *fur*, *ompA*, *oma87*, *plpB*, *hsf-2*, *nanH* and *hgbB*, suggesting the presence of these genes were broad characteristics of *P. multocida*. We also found approximately 63.92% (101/158), 51.27% (81/158), 8.86% (14/158), 7.59% (12/158), 3.16% (5/158), 0.63% (1/158), and 0.63% (1/158) of the isolates grew well in media with the presence of colistin (4 μg/mL), tetracycline (16 μg/mL), tigecycline (1 μg/mL), ampicillin (32 μg/mL), chloramphenicol (32 μg/mL), cefepime (16 μg/mL), and ciprofloxacin (1 μg/mL), respectively. This study contributes to the understanding of genotypes and antimicrobial resistance profile of *P. multocida* currently circulation in pigs of China.

**Supplementary Information:**

The online version contains supplementary material available at 10.1186/s44149-021-00031-7.

## Miantext

*Pasteurella multocida* is a multiple host gram-negative pathogen and a leading cause of pig respiratory disorders in the world (Peng et al. 2019a). Previously, two serotyping systems were developed based on the bacterial capsular antigens or the lipopolysaccharide (LPS) antigens which assigned *P. multocida* isolates into five serogroups (A, B, D, E and F) (Carter 1955) and/or 16 serovars (serovars 1 ~ 16), respectively (Heddleston et al. 1972). The application of these two serotyping systems in epidemiological studies has helped to address the serotypes of *P. multocida* circulating in different host species and their correlations to the diseases caused by the agent, thereby contributing to the vaccine development (Singh et al. 2014; Shivachandra et al. 2011; Dabo et al. 2007; Takashima et al. 2001). However, these two serological methods require high-quality antisera which are very difficult to be prepared and therefore it is not convenient their application in veterinary clinic (Peng et al. 2019a). Recently, the development of two multiplex PCR assays assigned the five capsular serogroups into five genotypes (A, B, D, E and F) (Townsend et al. 2001) and/or the 16 LPS serovars into eight LPS genotypes (L1 ~ L8) (Harper et al. 2015). Based on the outcomes of these two multiplex PCR assays, we previously have established a typing system to assign *P. multocida* isolates from different host species into capsular: LPS genotypes (Peng et al. 2019a; Peng et al. 2018a; Peng et al. 2018b; Lin et al. 2021), and we have determined that a capsular: LPS genotype D:L6 is the most prevalent types in pigs in China according to our molecular investigation on 115 *P. multocida* isolates from the lungs of pigs with respiratory disease in China in 2015 (Peng et al. 2018b). Now 5 years have passed by, considering the distribution and prevalence of *P. multocida* serotypes (or genotypes) may vary considerably over time in a given region (Tang et al. 2009), continuously investigating and monitoring *P. multocida* genotypes in pigs of China is meaningful for understanding the latest epidemiological profiles of *P. multocida* in Chinese pig farms, which is also beneficial for the development of effective vaccines against *P. multocida* infections. Therefore, we undertook a separate project to determine the current profiles of capsular: LPS genotypes of *P. multocida* isolates in pigs of China in recent years.

**Fig. 1:**
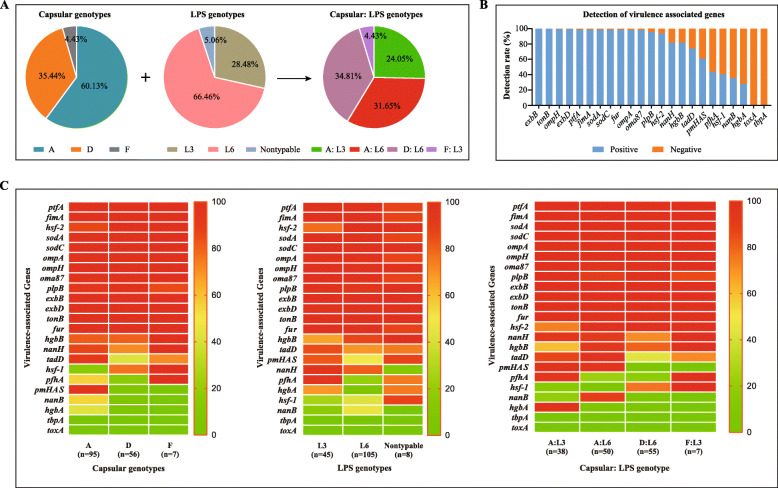
Genotypical characteristics of *P. multocida* isolates from pigs of China between 2019 and 2020. **A** Capsular and LPS genotypes of *P. multocida* isolates from pigs; **B** Detection rates of virulence factor-encoding genes of *P. multocida* isolates from pigs; **C** Distribution of different virulence factor-encoding genes between different capsular genotypes, LPS genotypes, or capsular: LPS genotypes

To understand the capsular genotypes and LPS genotypes of swine *P. multocida* prevalent in pig farms of China, we investigated 158 *P. multocida* isolates recovered from 1371 nasal swabs and/or lungs of pigs with respiratory disorders in pig farms from 16 provinces of China between September 1, 2019 and December 12, 2020 in this study. The results reveled that types A and D were main capsular genotypes for the 158 isolates, accounting for 60.13% (95/158) and 35.44% (56/158) of the total isolates, respectively (Fig. 1A). Only two LPS genotypes were determined: L3 and L6, accounting for 28.48% (45/158) and 66.46% (105/158), respectively (Fig. 1A). In addition, eight isolates (5.06%, 8/158) were found to be nontypable using the LPS genotyping method. When combining the capsular genotypes and the LPS genotypes, D: L6 (34.81%, 55/158) and A: L6 (31.65%, 50/158) were the predominant genotypes, followed by A: L3 (24.05%, 38/158) (Fig. 1A). These genotyping results are in agreement with those of our continuously monitoring of *P. multocida* isolates from pigs of China from 2013 to 2017 (Peng et al. 2018b; Peng et al. 2019b) as well as our genotypical characterizations of *P. multocida* from different host species through the whole genome sequences (Peng et al. 2019a; Peng et al. 2018a). In particular, *P. multocida* capsular: LPS genotypes D: L6 and A: L3 are also prevalent in pig herds in several other regions in the world (Yeh et al. 2017; Ujvári et al. 2019). These findings suggest *P. multocida* capsular: LPS genotypes D: L6 and A: L3 remain to be the main epidemic genotypes in pigs of China. The capsular and LPS genotyping results of swine *P. multocida* recovered between 2019 and 2020, together with our previous monitoring results of swine *P. multocida* recovered between 2013 and 2017 (Peng et al. 2018b; Peng et al. 2019b), these nearly 10-years monitoring results indicate *P. multocida* capsular: LPS genotypes D: L6 and A: L3 are prevalent in pigs of China. Based on these findings, we intend to develop a multivalent inactivated vaccine against *P. multocida* infections in pigs.

It has been reported that *P. multocida* possesses multiple virulence factors contributed to the fitness and pathogenesis (Harper et al. 2006), and a large proportion of genes participated in the synthesis of these virulence factors (Peng et al. 2019a; May et al. 2001; Peng et al. 2016). Therefore, virulence genotyping based on the detection of different virulence gene profiles has also been used as a useful genotyping method (Peng et al. 2019a; Devi et al. 2018; Massacci et al. 2018; Khamesipour et al. 2014). In this study, we detected 23 kinds of virulence factors encoding genes (VFGs) that are commonly targeted in *P. multocida* epidemiological studies (Peng et al. 2019a; Peng et al. 2018b; Khamesipour et al. 2014). The results revealed over 80% of the isolates were positive for *exbB* (100%; 158/158), *tonB* (100%; 158/158), *exbD* (99.37%; 157/158), *ompH* (99.37%; 157/158), *ptfA* (98.73%; 156/158), *fimA* (98.73%; 156/158), *sodA* (98.73%; 156/158), *sodC* (98.73%; 156/158), *fur* (98.73%; 156/158), *ompA* (98.10%; 155/158), *oma87* (98.10%; 155/158), *plpB* (95.57%; 151/158), *hsf-2* (93.67%; 148/158), *nanH* (81.65%; 129/158) and *hgbB* (81.65%; 129/158) (Fig. 1B). These VFGs encoding proteins participate in bacterial colonization and adherence (*ptfA*, *fimA* and *hsf-2*), iron uptake and acquisition (*exbB*, *tonB*, *exbD*, *fur* and *ompA*), stress-resistance and self-defense (*ompH*, *sodA*, *sodC, oma87*, *plpB*, *nanH* and *hgbB*), which are widely involved in the fitness and pathogenesis of *P. multocida* (Peng et al. 2019a; Harper et al. 2006). Therefore, the presence of these VFGs is proposed as a common characteristics of *P. multocida*, with no difference between strains from different hosts or different serotypes/genotypes (Peng et al. 2019a; Peng et al. 2018a; Smith et al. 2021; Furian et al. 2016). Less than 40% of the isolates were positive for *nanB* (35.44%; 56/158), *hgbA* (27.85%; 44/158), and in particular, no isolates were positive for *toxA* (0.00%; 0/158), and *tbpA* (0.00%; 0/158) (Fig. 1B). These results are also in agreement with those from the other studies (Peng et al. 2019b; Khamesipour et al. 2014; Smith et al. 2021). *toxA* encodes a 146-kDa single-chain G-protein-deamidating toxin (PMT), which is commonly associated with the progressive atrophic rhinitis (PAR) (Harper et al. 2006). *toxA*-positive isolates are not easily recovered from pigs without obvious clinical signs of PAR (Wilson and Ho 2013). *tbpA* encodes the transferrin-binding protein A (TbpA), which is an important iron-uptake receptor in many gram-negative bacteria (Pogoutse and Moraes 2017). Previous articles have documented that TbpA is only possessed by *P. multocida* isolates from bovine species and small ruminants (Harper et al. 2006; Ewers et al. 2006). There is no difference in the distribution of different types of VFGs between different capsular: LPS genotypes, with the exception of *hgbA*, *nanB*, *hsf-1*, *pfhA* and *pmHAS* (Fig. 1C).

As an emerging zoonotic pathogen, *P. multocida* may also pose a threat to public health (Register and Brockmeier 2019). Since administration of antimicrobials remains the first option for the treatment of *P. multocida* infections in both human and veterinary medicine (Wilson and Ho 2013), we tested the capacity of several kinds of antibiotics (ampicillin, imipenem, tetracycline, tigecycline, ciprofloxacin, cefepime, chloramphenicol, and colistin) commonly used in human or veterinary medicine on inhibiting *P. multocida*. To explore this, the 0.5 McFarland (Mc) concentration of the bacterial suspension was inoculated in Mueller-Hinton (MH) broth (Hopebio, Qingdao, China) with a final cell turbidity of 5 × 10^5^ CFU/ml. The broth was then plated on MH agars containing ampicillin (32 μg/m), imipenem (4 μg/mL), tetracycline (16 μg/mL), tigecycline (1 μg/mL), ciprofloxacin (1 μg/mL), cefepime (16 μg/mL), chloramphenicol (32 μg/mL), and/or colistin (4 μg/mL). After an incubation at 37 °C for 16 ~ 24 h we found 63.92% (101/158), 51.27% (81/158), 8.86% (14/158), 7.59% (12/158), 3.16% (5/158), 0.63% (1/158) and 0.63% (1/158) of the isolates grew well on the agars with the presence of colistin, tetracycline, tigecycline, ampicillin, chloramphenicol, cefepime and ciprofloxacin with the above concentrations, respectively. However, there was no *P. multocida* growing on MH agars containing 4 μg/mL of imipenem. Among the above antibiotics tested, the United States Clinical & Laboratory Standards Institute (CLSI) documents VET01-S and/or VET06 contain clinical breakpoints for ampicillin (Resistant≥16 μg/mL), tetracycline (Resistant≥8 μg/mL), and chloramphenicol (Resistant≥32 μg/mL). Based on these breakpoints, this study suggests that resistant rate of swine *P. multocida* is still high. This might be associated with a condition that tetracyclines are largely and commonly used in livestock of China (Yang et al. 2019). A notable finding in this research is that 63.92% (101/158) of the isolates grow well in the presence of 4 μg/mL colistin, and 8.86% (14/158) of the isolates grow well in the presence of 1 μg/mL tigecycline. Both colistin and tigecycline are recognized as the last resort antibiotics for the treatment of infections caused by multidrug resistant gram-negative bacteria (He et al. 2019; Liu et al. 2016). Although clinic breakpoints of these important antibiotics are not available for *P. multocida*, strains with similar phenotypes in *Enterobacteriaceae* are defined to be colistin-resistant (CLSI document M100, 31st Edition; https://clsi.org/standards/products/microbiology/documents/m100/) or tigecycline-resistant (EUCAST clinical breakpoints-bacteria (V. 11.0); https://www.eucast.org/fileadmin/src/media/PDFs/EUCAST_files/Breakpoint_tables/v_11.0_Breakpoint_Tables.pdf). In the next, we intend to measure the minimum inhibitory concentrations (MICs) of these antibiotics against *P. multocida* isolates. However, *P. multocida* isolates with high inhibitory concentrations against these important antibiotics should receive more attention.

To be concluded, we found *P. multocida* capsular: LPS genotypes D:L6 and A:L3 are still prevalent in pigs of China . In addition, we found a large proportion of *P. multocida* isolates could still grow in the presence of 4 μg/mL colistin, and a proportion of isolates could grow in the presence of 1 μg/mL tigecycline. These isolates should receive more attention because *Enterobacteriaceae* strains with similar phenotypes are defined as colistin- and tigecycline-resistant strains, and as a potential zoonotic pathogen, treatment of multidrug resistant *P. multocida* might face some challenge in human medical activities.

## Methods

### Sample collection and bacterial isolation

Between September 1, 2019 and December 30, 2020, a total of 1371 lung samples and nasal swabs of pigs with respiratory disorders were sent by pig farms from 16 Chinese provinces to the Veterinary Diagnostic Laboratory of Huazhong Agricultural University at Wuhan, China for *P. multocida* detection. *P. multocida* isolation, purification and identification was performed as described previously (Peng et al. 2018b). Each of the *P. multocida* isolates recovered was finally confirmed by PCR amplifying the species-specific *KMT1* gene (457 bp; Table S1 in supplementary materials) following the previously documented protocols (Townsend et al. 2001). Our previously typed swine *P. multocida* isolates HB03 (genotype A: L3, GenBank accession no. CP003328) (Peng et al. 2019a), HNA04 (genotype A: L6, GenBank accession no. PPVJ00000000) (Peng et al. 2018a), HN04 (genotype B: L2, GenBank accession no. PPVE00000000) (Peng et al. 2018a), HN06 (genotype D: L6, GenBank accession no. CP003313) (Liu et al. 2012), and HN07 (genotype F: L3, GenBank accession no. CP007040) (Peng et al. 2017) were used as quality-control strains in this study. Their genomic DNAs were extracted and were used as positive control for the PCR assays performed in this study.

### Capsular genotyping, LPS genotyping, and virulence genotyping

Capsular genotyping was performed using the multiplex PCR method described by Townsend et al. (Townsend et al. 2001). LPS genotyping was performed using the multiplex PCR method described by Harper et al. (Harper et al. 2015). Virulence genotyping was performed by PCR detection of 23 kinds of VFGs as described by Khamesipour et al. (Khamesipour et al. 2014). Primers for the genotyping of *P. multocida* are given in Table S1 in supplementary materials.

### Screening of *P. multocida* isolates growing on agars with specific concentrations of antibiotics

The capacity of several kinds of antibiotics (ampicillin, imipenem, tetracycline, tigecycline, ciprofloxacin, cefepime, chloramphenicol and colistin) commonly used in human or veterinary medicine on inhibiting *P. multocida* was tested following the guidelines for antimicrobial susceptibility testing published by CLSI (CLSI 2018), with several modifications. Briefly, single colonies were picked and suspended into sterile 0.9% normal saline to the concentration of 0.5 McFarland (Mc). The bacterial suspension was then inoculated in fresh Mueller-Hinton (MH) broth (Hopebio, Qingdao, China) with a final cell turbidity of 5 × 10^5^ CFU/mL. Afterwards, 100 μl of the broth was plated on MH agars containing ampicillin (32 μg/mL; MedChemExpress [MCE], US), imipenem (4 μg/mL; MCE, US), tetracycline (16 μg/mL; MCE, US), tigecycline (1 μg/mL; MCE, US), ciprofloxacin (1 μg/mL; MCE, US), cefepime (16 μg/mL; MCE, US), chloramphenicol (32 μg/mL; MCE, US), and/or colistin (4 μg/mL; MCE, US). The plates were finally incubated at 37 °C for 16 ~ 24 h to observe the growth condition of the bacterium. *E. coli* ATCC®^*^ 25922 was used as quality control. 

## Supplementary Information

**Additional file 1: Table S1.** Primers used for *P. multocida*genotyping.

## Data Availability

Not applicable.

## Abbreviations

ARG: Antimicrobial resistance gene
LPS: Lipopolysaccharide
VFG: Virulence factors encoding gene
