# Supplementary material for: Pasteurella multocida capsular: lipopolysaccharide types D:L6 and A:L3 remain to be the main epidemic genotypes of pigs in China
Source: Anim Dis. 2021 Nov 2;1(1):26. doi: 10.1186/s44149-021-00031-7 (PMC8561366; doi:10.1186/s44149-021-00031-7)
Supplement: Supplementary file 1 — Additional file 1: Table S1. Primers used for P. multocidagenotyping. [file 44149_2021_31_MOESM1_ESM.docx]

Table S1. Primers used for *P. multocida* genotyping.

| **Genotyping** | **Primers** | **Sequences (5’-3’)** | **Product size (bp)** | **Annealing temperature (°C)** | **Purpose** |
| --- | --- | --- | --- | --- | --- |
| *P. multocida* species specific gene | Pm-F | ATCCGCTATTTACCCAGTGG | 457 | 56 | *P. multocida* identification |
|  | Pm-R | GCTGTAAACGAACTCGCCAC |  |  |  |
| Capsular genotyping | PmA-F | GATGCCAAAATCGCAGTCAG | 1048 | 56 | Capsular genotype A |
|  | PmA-R | TGTTGCCATCATTGTCAGTG |  |  |  |
|  | PmB-F | CATTTATCCAAGCTCCACC | 758 | 56 | Capsular genotype B |
|  | PmB-R | GCCCGAGAGTTTCAATCC |  |  |  |
|  | PmD-F | TTACAAAAGAAAGACTAGGAGCCC | 647 | 56 | Capsular genotype D |
|  | PmD-R | CATCTACCCACTCAACCATATCAG |  |  |  |
|  | PmE-F | TCCGCAGAA AATTATTGACTC | 512 | 56 | Capsular genotype E |
|  | PmE-R | GCTTGCTGCTTGATTTTGTC |  |  |  |
|  | PmF-F | TCGGAGAACGCAGAAATCAG | 852 | 56 | Capsular genotype F |
|  | PmF-R | TTCCGCCGTCAATTACTCTG |  |  |  |
| LPS genotyping | L1-F | ACATTCCAGATAATACACCCG | 1307 | 54 | LPS genotype L1 |
|  | L1-R | ATTGGAGCACCTAGTAACCC |  |  |  |
|  | L2-F | CTTAAAGTAACACTCGCTATTGC | 810 | 54 | LPS genotype L2 |
|  | L2-R | TTTGATTTCCCTTGGGATAGC |  |  |  |
|  | L3-F | TGCAGGCGAGAGTTGATAAACCATC | 474 | 54 | LPS genotype L3 |
|  | L3-R | CAAAGATTGGTTCCAAATCTGAATGGA |  |  |  |
|  | L4-F | TTTCCATAGATTAGCAATGCCG | 550 | 54 | LPS genotype L4 |
|  | L4-R | CTTTATTTGGTCTTTATATATACC |  |  |  |

|  | L5-F | AGATTGCATGGCGAAATGGC | 1175 | 54 | LPS genotype L5 |
| --- | --- | --- | --- | --- | --- |
|  | L5-R | CAATCCTCGTAAGACCCCC |  |  |  |
|  | L6-F | TCTTTATAATTATACTCTCCCAAGG | 668 | 54 | LPS genotype L6 |
|  | L6-R | AATGAAGGTTTAAAAGAGATAGCTGGAG |  |  |  |
|  | L7-F | CCTATATTTATATCTCCTCCCC | 931 | 54 | LPS genotype L7 |
|  | L7-R | CTAATATATAAACCATCCAACGC |  |  |  |
|  | L8-F | GAGAGTTACAAAAATGATCGGC | 255 | 54 | LPS genotype L8 |
|  | L8-R | TCCTGGTTCATATATAGGTAGG |  |  |  |
| Virulence genotyping | PtfA-F | TGTGGAATTCAGCATTTTAGTGTGTC | 488 | 55 | Type 4 fimbriae |
|  | PtfA-R | TCATGAATTCTTATGCGCAAAATCCTGCTGG |  |  |  |
|  | FimA-F | CCATCGGATCTAAACGACCTA | 866 | 55 | Fimbriae |
|  | FimA-R | AGTATTAGTTCCTGCGGGTG |  |  |  |
|  | Hsf_1-F | TTGAGTCGGCTGTAGAGTTCG | 654 | 55 | Autotransporter adhesion |
|  | Hsf_1-R | ACTCTTTAGCAGTGGGGACAACCTC |  |  |  |
|  | Hsf_2-F | ACCGCAACCATGCTCTTAC | 433 | 55 | Autotransporter adhesion |
|  | Hsf_2-R | TGACTGACATCGGCGGTAC |  |  |  |
|  | PfhA-F | TTCAGAGGGATCAATCTTCG | 286 | 55 | Filamentous hemagglutinin |
|  | PfhA-R | AACTCCAGTTGGTTTGTCG |  |  |  |
|  | TadD-F | TCTACCCATTCTCAGCAAGGC | 416 | 55 | Putative nonspecific tight adherence protein D |
|  | TadD-R | ATCATTTCGGGCATTCACC |  |  |  |
|  | ToxA-F | CTTAGATGAGCGACAAGG | 864 | 55 | Dermonecrotic toxin |
|  | ToxA-R | GAATGCCACACCTCTATAG |  |  |  |
|  | ExbB-F | TTGGCTTGTGATTGAACGC | 283 | 55 | Accessory protein Ton-dependent transport of iron compounds |
|  | ExbB-R | TGCAGGAATGGCGACTAAA |  |  |  |
|  | ExbD-F | CGTTCTGATTACAGCCTCTT | 247 | 55 | Accessory protein Ton-dependent transport of iron compounds |
|  | ExbD-R | AACGAAATCTTGGAAACTGG |  |  |  |
|  | TonB-F | CGACGGTGAAACCTGAGCCA | 261 | 55 | Iron transporters, transport ferric-siderophore complexes |
|  | TonB-R | CCGAGCGATAAGCATTGACT |  |  |  |
|  | HgbA-F | TCAACGGCAGATAATCAGGG | 267 | 55 | Hemoglobin-binding protein A |
|  | HgbA-R | GCGGGAATGCTGAAGATAAG |  |  |  |
|  | HgbB-F | GTTTACCGTGTATTAGACCA | 244 | 55 | Hemoglobin-binding protein B |
|  | HgbB-R | CATTACTACATTTGCCATAC |  |  |  |
|  | Fur-F | ACCGCGTTGGAATTATGATTG | 788 | 55 | Ferric uptake regulation protein |
|  | Fur-R | CATTGAGTACGGCTTGACAT |  |  |  |
|  | TbpA-F | TTGGTTGGAAACGGTAAAGC | 728 | 55 | Transferrin-binding protein A |
|  | TbpA-R | TAACGTGTACGGAAAAGCCC |  |  |  |
|  | NanB-F | CATTGCACCTAACACCTCT | 555 | 55 | Outer membrane-associated proteins, an autotransporter  protein |
|  | NanB-R | GGACACTGATTGCCCTGAA |  |  |  |
|  | NanH-F | GTGGGAACGGGAATTGTGA | 287 | 55 | Outer membrane-associated proteins, small sialidases |
|  | NanH-R | ACATGCCAAGTTTGCCCTA |  |  |  |
|  | pmHA-F | TCAATGTTTGCGATAGTCCGTTAG | 430 | 55 | Hyaluronan synthase |
|  | pmHA-R | TGGCGAATGATCGGTGATAGA |  |  |  |
|  | OmpA-F | CGCATAGCACTCAAGTTTCTCC | 201 | 55 | Outer membrane protein A |
|  | OmpA-R | CATAAACAGATTGACCGAAACG |  |  |  |
|  | OmpH-F | CGCGTATGAAGGTTTAGGT | 438 | 55 | Outer membrane protein H |
|  | OmpH-R | TTTAGATTGTGCGTAGTCAAC |  |  |  |
|  | Oma87-F | GGCAGCGAGCAACAGATAACG | 838 | 55 | Outer membrane protein 87 |
|  | Oma87-R | TGTTCGTCAAATGTCGGGTGA |  |  |  |
|  | PlpB-F | TTTGGTGGTGCGTATGTCTTCT | 282 | 55 | Lipoprotein B |
|  | PlpB-R | AGTCACTTTAGATTGTGCGTAG |  |  |  |
|  | SodA-F | TACCAGAATTAGGCTACGC | 361 | 55 | Superoxide dismutase A |
|  | SodA-R | GAAACGGGTTGCTGCCGCT |  |  |  |
|  | SodC-F | AGTTAGTAGCGGGGTTGGCA | 235 | 55 | Superoxide dismutase B |
|  | SodC-R | TGGTGCTGGGTGATCATCATG |  |  |  |
